# The Diseases of the Pulp

**Published:** 1909-08-15

**Authors:** Russell W. Bunting

**Affiliations:** Ann Arbor, Mich.


### THE DISEASES OF THE PULP.

BY RUSSELL W. BUNTING, D.D.SC., ANN ARBOR, MICH.

In connection with the consideration of the diseases of the pulp, it will be well to review, briefly, the structure of that organ, and consider some of its characteristics. The pulp is the remains of the dentinal papilla, which persists in the full formed tooth as a medium of nutrition and sensation. The tissue of which it is composed, remains in the embryonic state, while in health, and is surrounded by a continuous layer of columnar cells, the odontoblasts. This odontoblastic layer is in intimate association with the dentine, the fibrillar continuations of the odontoblasts lying in the dentinal canals, and throughout their length extending to the enamel dentine junction.

The pulp contains blood vessels and nerves of a modified type. The blood vessels enter the apical foramine in one or two trunks, and run through the body of the pulp to end in a free capillary plexus just beneath the layer of odontoblasts. From the capillaries the blood is collected by the veins and carried out through the apical foramen. These blood vessels are peculiar in that they have little or no muscle in their walls, very often being bounded by only a single layer of endothelium.

The nerves entering the pulp are of two kinds sensory and sympathetic. The sensory type end in small knobs or granules between the odontoblasts, or between the odontoblasts and dentine—they are not in direct contact with the odontoblasts nor do they enter the dentine. The sympathetic nerves are vasomotor in their function and end on the walls of the blood vessels. The nerves of the pulp are characteristic, in that they loose their medullary sheaths on entering the apical foramen.

The diseases of the pulp are acute and chronic, depending upon the character and duration of the causative factor. The pulp diseases are divided according to their morphology into two classes, those which are constructive and those which are destructive. In the former class occur those pathological changes in the pulp which are accompanied by a formation of atypical growths, such as calcarious deposits, and hypertrophies, while in the latter we have those changes which are strictly degenerative in character, and tend toward the death and destruction of the pulp.

### THE CONSTRUCTIVE DISEASES OF THE PULP.

The simplest form of disease of this type, is a change which takes place in the dentine due to the activity of the odontoblasts of the pulp. This change usually occurs in certain areas of the dentine which are affected by some outside irritation, such as abrasion, erosion, and caries. The area affected is spoken of as "The Transparent Zone," and consists in a consolidation of the dentinal tubules in that area. This is probably accomplished by either a mechanical or chemical irritation of the ends of the dentinal fibrillae, which stimulates the odontoblasts, of which they are a prolongation, to activity. This odontoblastic activity, especially under slow forms of stimulation, will often deposit calcarious material in the dentinal fibrillae, causing a consolidation of the canals and fibrils so that the dentine in that area, instead of being a porous substance, is a solid calcarious mass. Under the microscope this area has a homogeneous appearance and more translucent than the surrounding dentine, hence the name "Transparent Zone." This consolidation of the dentine has the office of protecting the tooth and pulp in some measure, against invading forces, such as erosion abrasion and caries, and are only formed by those pulps which are in good health, and under the influence of slow stimuli.

Associated with the formation of the "Transparent Zone" and deposited under similar conditions, is the growth of

Secondary Dentine in the pulp. This deposition, like the former, is the result of the irritation of the odontoblasts by mechanical, chemical or thermal stimuli, acting on the ends of the dentinal fibrilli, which under certain circumstances causes the odontoblasts to take up again their work of forming dentine which they had ceased when the tooth was fully formed. A great range in the structure of this new dentine is seen, depending upon the health and activity of the pulp. There is in the formation a deposition of calcarious material, as in the primary dentine, and a variable number of dentinal fibrils are included in the mass. In those which are formed by healthy pulps and under a mild form of irritation, the new dentine may have an equal number of tubules and a structure similar to that of the old—this difference, however, obtains, the direction of the tubules in the new dentine is always at an angle to the direction of those in the primary dentine. This well formed secondary dentine, which so closely approximates the normal, is only found in the first few layers formed, and if the deposition goes on to any great extent the number of tubules will diminish, and those which do remain will be very much curved and contorted. very often, when a large mass has been deposited, the structure will appear amorphous and will include remains of the pulp tissue. Secondary dentine may be in the form of a thin layer on the pulpal wall of the primary dentine, at a point corresponding with the location of the injury, it may be in the form of a nodule, or bunch extending out into the pulp from one of the walls, it may entirely fill one horn of the pulp chamber, or may extend down into the canals of the roots, partially or completely occluding them. Secondary dentine is formed under abrasion, erosion, caries, pulp cappings, gold crowns, etc., but more especially under abrasion. Its office is to protect the pulp from exposure by the wear of the tooth, and from the irritation of excessive thermal or chemical stimuli. Its disadvantages are that it may seriously interfere with the circulation of the pulp, either by irritation or strangulation of the tissue, and in many instances it seriously affects the removal of the pulp from the root canals.

Under certain kinds of irritation, the pulp undergoes a form of calcific degeneration. The cells of the pulp die in certain areas, and in and about the dead areas, calcific material is deposited by the surrounding pulp. This calcarious matter is first deposited in the form of unfused globules, which later unite into a mass and harden. In this manner, in some conditions, small nodules of calcific matter may be formed in any region of the pulp. These nodules may be of any shape or size, they are nearly translucent in appearance, and are usually composed of concentric layers of calcified material about a central nidus of dead organic substance. In the mass there may be included a variable amount of organic matter, remains of dead pulp cells, and in some forms there are tubes and fibrils running throughout, without any definate arrangement. Under a continuance of the irritation, a large number of these small concretions may form and uniting, give rise to a large mass of calcarious material. These larger masses are known as "Pulp Nodules," or "Pulp Stones" and are usually formed in the pulp chamber. They are seldom attached to the walls of the pulp chamber, but, being formed in the substance of the pulp, they are probably kept from uniting with the wall by the gurgitation of the blood about them. Any size of nodule may be formed, from the very small spicules and granules, to those which practically fill the pulp chamber. Pulp nodules occur, not only in the pulp chamber, but are found occasionally in the root canals. Here they may be free in the substance of the pulp tissue, or as is sometimes seen, they are imbedded in the wall of the canal and protrude out into the lumen of the canal a considerable distance. In such instances they probably replace a portion of the wall of the tubule which has been resorbed by the pulp, and in the replacement, the calcarious and nodule-like material is deposited in excess, so that it narrows the lumen, and in some cases almost closes it.

Pulp stones are formed under the stimulus of an external irritant, such as abrasion, erosion, caries, fillings, gold crowns, etc. The pulp stones are not always formed in the teeth affected, but, by reflex stimuli, may form in a tooth adjacent to the one which is being irritated. Such formations are in no sense a remedial measure, but are simply a calcific degeneration, and tend toward the death and destruction of the pulp. By their form and bulk they offer a variable amount of interference to the circulation, giving rise to thrombosis, passive hyperaemia, etc. At the same time they press upon the sensory nerves of the pulp, causing pains, which may be located in the tooth or referred some distance, manifesting itself in a very hidden neuralgia. These concretions also offer considerable difficulty in the removal of the pulp—the large ones in the chamber, make it difficult to apply drugs to the living portion beneath them, and the small ones in the canals may prevent the removal of the pulp tissue beyond them.

In addition to the formation of distinct nodules or spherules of calcarious material in the pulp, there is also seen a condition which is analagous to the ordinary calcific degeneration, occurring in any part of the body. There is usually a loss of the cellular elements of the pulp and a development of an excess of fibrils, which precedes the deposition of lime salts. The salts are deposited about the dead cells and fibrils, forming small granular masses, throughout the pulp. Upon removing such a pulp, it can be withdrawn very easily from the canals, and may be more or less stiff and granular due to the calcific deposits. This condition is induced by the presence of secondary dentine, pulp stones, and other continued irritations which tend to interfere with the circulation of the pulp.

In general the constructive changes in the pulp are induced by the slower forms of irritation, such as slow caries, slow erosion, and especially by abrasion. Under abrasion, pulps will often calcify the whole bulbal portion, and occasionally the entire chamber and root-canals are obliterated. Calcifications undoubtedly form under many of the filling materials which are inserted for the repair of caries, due to their irritation. In the case of metallic fillings the irritation is that of heat and cold, which is readily transmitted through them, while in the case of cements there is the irritation of the acid component. Very often a pulp is so irritated by the filling that there is considerable pain produced for a time after the introduction, but, if the pulp is healthy, it may build sufficient calcific material to protect itself from the thermal changes, and the action of the acids.

All of the constructive changes are a menace to the life and health of the pulp, and may produce pains which are reflected some distance, setting up a neuralgia, which is exceedingly hard to diagnose. There is a further disadvantage, in that all of the solid calcarious deposits tend to render their extirpation of the pulp much more difficult.

